# Parental knowledge and satisfaction with newborn hearing screening in Saudi Arabia

**DOI:** 10.3389/fped.2024.1464697

**Published:** 2024-11-20

**Authors:** Noura I. Alothman, Ahmad A. Alanazi, Sadeem S. Alshenaifi, Yara Alhadhban, Salma A. Alateeq, Norah Alhwaimel, Nouf Dolgum

**Affiliations:** ^1^ Department of Health Communication Sciences, College of Health and Rehabilitation Sciences, Princess Nourah bint Abdulrahman University, Riyadh, Saudi Arabia; ^2^ Department of Audiology and Speech Pathology, College of Applied Medical Sciences, King Saud bin Abdulaziz University for Health Sciences, Riyadh, Saudi Arabia; ^3^ King Abdullah International Medical Research Center, Riyadh, Saudi Arabia

**Keywords:** hearing screening, newborns, knowledge, satisfaction, questionnaire, Saudi Arabia

## Abstract

**Objectives:**

Parental knowledge and satisfaction are critical for evaluating the newborn hearing screening (NHS) program. No studies have assessed both parental knowledge and satisfaction with the NHS program since its launch in Saudi Arabia. The study aimed to evaluate parents' knowledge and satisfaction with the NHS program in Saudi Arabia.

**Methods:**

This study included parents of children aged 3 years or younger who had undergone NHS. A questionnaire with 23 questions in Arabic assessed knowledge and satisfaction. Knowledge-related questions were developed by a focus group, while satisfaction-related questions were derived from the Parent Satisfaction Questionnaire with a Neonatal Hearing Screening Program.

**Results:**

A total of 796 parents participated. Overall, 92.8% reported that their newborns passed the screening, 1.6% reported that their newborns failed, and 5% were unaware of the NHS results. About 0.6% did not return for follow-up appointments due to unawareness of the need for follow-up. Only 29.6% received information about the NHS before the screening, and 44.7% reported receiving sufficient information about the results. Most parents (80.2%) were generally satisfied with the NHS program.

**Conclusions:**

Parents in this study were generally knowledgeable and satisfied with the NHS program. However, efforts are needed to improve the NHS program's effectiveness by increasing awareness and providing sufficient information on the necessity of NHS, test results, and follow-up appointments.

## 1 Introduction

Hearing impairment is an invisible disability that ranges from mild hearing loss to total deafness (1). Globally, the World Health Organization estimates that 1.5 billion individuals live with varying degrees of hearing impairment, with 34 million children requiring hearing rehabilitation (2). Hearing loss significantly impacts children's communication skills, academic achievement, and social interactions, often leading to feelings of loneliness, frustration, and low self-esteem (3–5). Globally, untreated hearing loss costs over $980 billion each year, encompassing healthcare, educational expenses, lost productivity, and various societal costs (6). Consequently, early hearing detection and intervention (EHDI) programs aim to identify and manage hearing loss early, thereby minimizing its negative effects (7, 8). Guidelines recommend screening all newborns for hearing within 1 month of birth, diagnosing hearing loss by 3 months, and providing intervention by 6 months (8). In Saudi Arabia, the Ministry of Health has implemented its mandatory Newborn Hearing Screening (NHS) program since 2016. The program requires all birth hospitals around the country to conduct hearing screening for all newborns, ensuring early detection and intervention for hearing impairments (9).

Parents are integral to the family-centered care approach and are crucial stakeholders in EHDI programs (10, 11). Despite this, many parents may lack knowledge about hearing loss and its implications, often feeling unprepared to decide on communication methods, hearing aids, and educational settings when their child is diagnosed with hearing loss (12, 13). A common challenge for NHS programs is parental refusal due to limited awareness of its importance and associated costs (14). For instance, only 22% of parents in the United States know the next step after their child is diagnosed with hearing loss (15). The educational background, attitudes, and support of parents significantly influence the outcomes of children with hearing loss (16). Furthermore, parental satisfaction serves as a central metric for evaluating the efficacy of screening programs in pediatric settings (17). Satisfied parents will collaborate better, adhere to their child's treatment, and attend follow-up sessions, regardless of screening results (18, 19). Therefore, parental knowledge and satisfaction are pivotal for achieving NHS program goals.

Previous studies have examined parental attitudes, satisfaction levels, and knowledge about NHS programs, revealing varied attitudes and satisfaction levels (18–24). However, few studies have explored these aspects in Saudi Arabia post-NHS program implementation. Alanazi investigated the referral and loss to follow-up (LTF) rates among 20,171 newborns in Riyadh, identifying parental awareness as a major contributor to high LTF rates (25). Alothman et al. measured the LTF rate in a Riyadh hospital, citing inadequate knowledge about follow-up and the NHS's overall importance as primary reasons (26). Almatrafi et al. studied predictors of parental recall regarding the NHS in Saudi Arabia, highlighting inadequate parental awareness despite recall biases (27). No studies have comprehensively measured both parental knowledge and satisfaction with the NHS program in Saudi Arabia. This study aims to assess parental knowledge and satisfaction with the NHS program.

## 2 Materials and methods

This cross-sectional descriptive study was conducted after receiving approval from the Institutional Review Board of Princess Nourah bint Abdulrahman University (Approval Number: 23-0767).

### 2.1 Study population

The study assessed parental knowledge and satisfaction regarding the NHS program among the parents of children aged three years or younger across all regions of Saudi Arabia. Data collection took place between November 2023 and February 2024. The study focused on the age group because the NHS program, initially implemented in Saudi Arabia's 30 hospitals in 2016, saw significant expansion and improvement across nearly all hospitals after 2021 (9).

### 2.2 Instrumentation

Data were gathered using an electronic questionnaire. Given the absence of standardized tools for assessing parental knowledge of the NHS program, questions related to knowledge were developed by a focus group. The satisfaction section utilized questions adapted from the Parent Satisfaction Questionnaire with Neonatal Hearing Screening Program, a validated instrument widely used in various countries, including Malaysia (11), Australia (18), Iran (19), Spain (23), and the United Kingdom (28). The Arabic version, validated in Jordan (24), was used with permission. The questionnaire underwent reviews by external experts to ensure content appropriateness and underwent pilot testing with five parents to confirm clarity and comprehension.

The final questionnaire comprised 23 Arabic-language closed-ended questions organized into three sections: parental demographics, knowledge, and satisfaction. The demographic section included 10 questions about parental and child details, such as age, education, hospital of birth, and presence of risk factors. The knowledge section included six questions about the child's NHS participation, screening outcomes, reasons for non-participation if applicable, whether there was a follow-up screening, what were the follow-up screening results, and reasons for not following up if applicable. The satisfaction section contained seven questions about the receipt and clarity of pre-screening information and post-screening results, communication with NHS personnel, and overall satisfaction with the program.

The electronic questionnaire link was distributed via social media platforms (e.g., Twitter, Facebook, and WhatsApp) and personal networks, including family and acquaintances. Participants were presented with a study information sheet and consent form on the first page of the questionnaire, allowing them to opt in or decline participation before proceeding with the questions.

## 3 Results

Data were collected from 1,087 respondents who completed the questionnaire. Among them, 796 (73.3%) participants had newborns who underwent the NHS, whereas 82 (7.5%) reported no experience with the NHS, and 209 (19.2%) were uncertain. Reasons cited for not undergoing the NHS included the coronavirus disease pandemic (36.6%), lack of knowledge about the screening (23.2%), and facility-related issues, such as equipment shortages and appointment unavailability (19.5%).

### 3.1 Demographics of the participants

Awareness of the NHS was assessed among participants (*n* = 796; 749 women; 47 men) with prior NHS experience. The majority were Saudi nationals (98%) aged 31–40 years, with fathers being slightly older than mothers. Most parents held a bachelor's degree (60.75%), with a small proportion of them having education levels lower than high schools (2.25%). Families typically had 1–3 children (57.8%), with a significant representation from the central region (68.5%), followed by the northern (10%), eastern (7.4%), and southern (7.3%) regions. NHS screenings occurred predominantly in private (56.7%) and government (43.3%) hospitals. Notably, 295 participants (37.1%) reported that their newborns had risk factors for hearing loss (Table 1).

**Table 1:** Demographic data of the participants.

Variables	*N* = 796
	*n* (%)
Gender	- Male: 47 (6%) - Female: 749 (94%)
Nationality	- Saudi: 780 (98%) - Non-Saudi: 16 (2%)
Residential area	- Central region: 545 (68.5%) - Eastern region: 59 (7.4%) - Western region: 54 (6.8%) - Northern region: 80 (10%) - Southern region: 58 (7.3%)
Father's age (years)	- Less than 20: 17 (2.1%) - Between 21 and 30: 92 (11.6%) - Between 31 and 40: 482 (60.6%) - Between 41 and 50: 189 (23.7%) - More than 50: 16 (2%)
Mother's age (years)	- Less than 20: 5 (0.6%) - Between 21 and 30: 305 (38.3) - Between 31 and 40: 416 (52.3%) - Between 41 and 50: 67 (8.4%) - More than 50: 3 (0.4%)
Father's educational level	- Less than high school: 17 (2.1%) - High school diploma or equivalent: 137 (17.2%) - Diploma degree: 75 (9.4%) - Bachelor's degree: 416 (52.3%) - Master's degree: 120 (15.1%) - PhD degree: 31 (3.9%)
Mother's educational level	- Less than high school: 19 (2.4%) - High school diploma or equivalent: 75 (9.4%) - Diploma degree: 64 (8%) - Bachelor's degree: 551 (69.2%) - Master's degree: 80 (10.1%) - PhD degree: 7 (0.9%)
Number of siblings	- No siblings: 210 (26.4%) - Between 1 and 3 = 460 (57.8%) - Between 4 and 6 = 115 (14.4%) - More than 7: *n* = 11 (1.4%)
Type of newborn birth facility	- Private hospital: 451 (56.7%) - Government hospital: 345 (43.3%)
Risk factors for hearing loss among newborns	- Yes: 295 (37.1%) - No: 501 (62.9%)

### 3.2 Knowledge about the NHS

**Figure 1:**
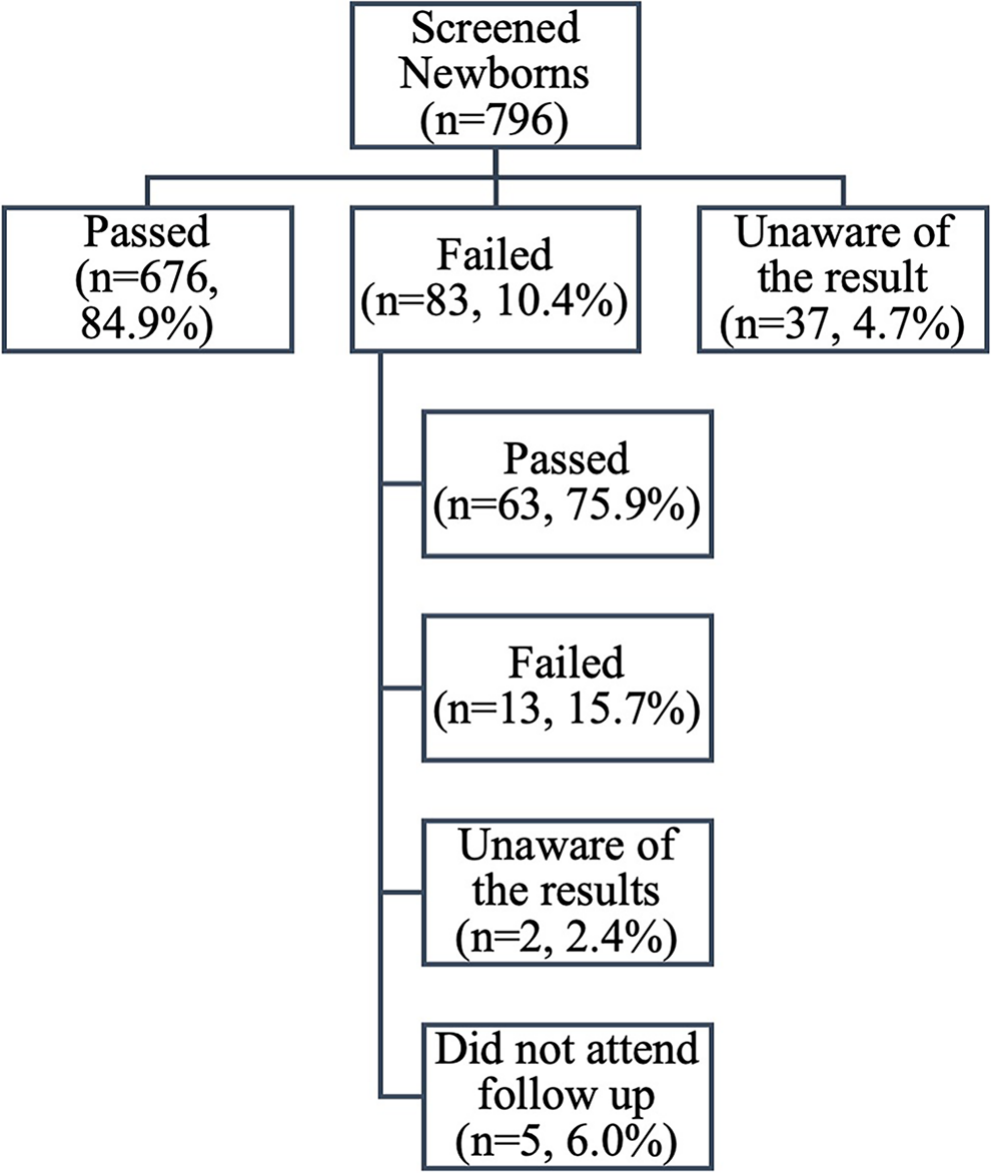
The results of newborn hearing screening and follow-up appointments.

Figure 1 summarizes the findings from participants whose newborns underwent the NHS (*n* = 796). Screening results indicated that 676 newborns (84.9%) initially passed, 83 newborns (10.4%) initially failed, and the parents of 37 newborns (4.6%) were unaware of their results. Among those who failed, 78 returned for follow-up; 63 (75.9%) passed, 13 (15.7%) failed, and the parents of 2 newborns (2.4%) were unaware of their results. Five (6.0%) did not attend follow-up due to unawareness of appointment availability. Overall, at the end of the screening, 92.8% of parents reported that their newborns passed, 1.6% reported that their newborns failed, 5% were unaware of their newborns' results, and 0.6% were lost to follow-up.

### 3.3 Satisfaction with the NHS

Participants with prior experience (*n* = 796) reported satisfaction levels, shown in Table 2. Only 236 (29.6%) received pre-screening information; 519 (65.2%) did not. Information was mainly delivered verbally to examiners (71.6%), through relatives (16.5%), printed materials (8.9%), and social media (3%). Of those informed, 193 (81.8%) participants found the information adequate, whereas 20 (8.5%) found it challenging.

**Table 2:** The results of parents’ satisfaction with the NHS.

Items	*N* = 796
	*n* (%)
Did you receive information about the newborn hearing screening program before implemented on your child?	- Yes: 236 (29.6%) - No: 519 (65.2%) - I do not know: 41 (5.2%)
How did you receive this information?	- Verbally by the examiner: 169 (71.6%) - Family, relatives or friends: 39 (16.5%) - Printed materials, such as brochures: 21 (8.9%) - Social media: 7 (3%)
Was the information provided to you about the newborn hearing screening program difficult to understand?	- Yes: 20 (8.5%) - No: 216 (91.5%)
Was the information provided to you about the newborn hearing screening program sufficient and useful?	- Yes: 193 (81.8%) - No: 43 (18.2%)
Did you receive sufficient information about the results of your child's hearing screening?	- Yes: 356 (44.7%) - No: 440 (55.3%)
Was the communication with the examiner easy?	- Yes: 558 (70.1%) - No: 238 (29.9%)
Are you satisfied with the newborn hearing screening program in the hospital where your child was born?	- Yes: 638 (80.2%) - No: 158 (19.8%)

Regarding post-screening results, 356 (44.7%) felt inadequately informed, and 558 (70.1%) found communication with examiners straightforward. Overall, 638 (80.2%) participants expressed satisfaction with the NHS program.

## 4 Discussion

The current study aimed to assess parental knowledge and satisfaction with the NHS program in Saudi Arabia. The study revealed that a majority of parents were aware of and generally satisfied with the NHS program. However, certain issues require further investigation and intervention to maximize the program's effectiveness.

### 4.1 Knowledge about the NHS

In this study, 73.3% of parents reported that their newborns had undergone the NHS, whereas 7.5% had not, and 19.2% were uncertain. The primary reason cited for not participating in the NHS was the coronavirus disease pandemic (36.6%), underscoring its impact on healthcare delivery (29). Additionally, 23.2% of parents mentioned a lack of knowledge about the NHS as a deterrent, possibly influenced by recall bias or insufficient information dissemination. The uncertainty rate (19.2%) was notably higher than that reported in similar programs, such as that in Malaysia (0%) (11).

Among parents whose newborns underwent the NHS, 92.8% reported their newborns passing the screening, 1.6% reported their newborns failing, and 5% were unaware of the results. The latter could be attributed to recall bias or inadequate result communication, contributing to uncertainty. The uncertainty rate in this study from Saudi Arabia was lower compared with those in studies from Malaysia (7.5%) (11), Iran (8.7%) (19), and Jordan (49%) (24). Notably, 0.6% of parents did not attend follow-up appointments due to unawareness, a common issue reported previously (26). In Saudi Arabia, there are a few awareness campaigns regarding the significance of hearing screening and the negative impacts of hearing loss (25).

Overall, addressing parental awareness gaps is crucial for successful NHS implementation (30). The Ministry of Health should consider supporting more public awareness campaigns in Saudi Arabia. The Joint Committee on Infant Hearing advised providing clear, written information to parents about the significance of NHS, screening outcomes, and follow-up steps to reduce LTF rates (8).

### 4.2 Satisfaction with the NHS

The study reported that 80.2% of parents were generally satisfied with the NHS program, similar to the rate in Malaysia (80.6%) (11). However, satisfaction levels were lower than those in Australia, Iran, and Jordan, where over 90% of parents expressed satisfaction (18, 19, 24).

The lower satisfaction in our study could be due to the inadequate pre- and post-NHS information. Only 29.6% received NHS information before screening, verbally or in writing, compared with higher rates in Malaysia (95%) (11) and Jordan (69.5%) (24). Conversely, 8.5% found the information difficult to understand, which is higher than those in other studies (0%–2.9%) (11, 24). Providing written, easily understandable NHS information alongside verbal explanations enhances parental understanding and adherence (14, 31). Timing of information delivery is crucial; most parents receive information post-birth (31, 32).

Regarding satisfaction with post-NHS information adequacy, over half of parents were dissatisfied with test result information availability, akin to Jordan (57.2%) (24) but higher than the proportion in Malaysia (26%) (11). Communication ease with examiners was challenging for 29.9% of parents, emphasizing the need for simple, jargon-free communication and comprehensive written materials.

Overall, parents were generally aware of and satisfied with the NHS program, and issues with program recognition and comprehension persist. Addressing these can enhance NHS program effectiveness in Saudi Arabia.

### 4.3 Study limitations and future research

This study has some limitations. Despite this study encompassing all regions of Saudi Arabia, most respondents (68.5%) were from the central region, warranting further regional studies. Moreover, integrating open-ended could unearth unconsidered issues. Future research could explore relationships between awareness, satisfaction, and parental demographics. The questionnaire included questions designed to answer the aim of the study; however, a detailed questionnaire is advised to be developed for obtaining comprehensive information about the parents' knowledge and satisfaction with the NHS program in Saudi Arabia.

## 5 Conclusion

Parental awareness and the NHS program in Saudi Arabia are positive, yet improvements in recognition and results comprehension are imperative. Enhanced awareness campaigns and accessible information provision could optimize NHS program outcomes.

## Data Availability

The datasets presented in this article are not readily available because data confidentiality. Requests to access the datasets should be directed to nialothman@pnu.edu.sa.
